# Supply chain management curriculum integration in pre-service training in Tanzania

**DOI:** 10.1186/2052-3211-7-S1-P14

**Published:** 2014-12-17

**Authors:** Matiko Machagge, Dorothy Matoyo, Irene Alenga

**Affiliations:** 1 SCMS & USAID|DELIVER PROJECT, John Snow Inc. (JSI), Dodoma, Tanzania

## Background

An assessment in 2011 identified the need to incorporate supply chain management (SCM) in academic curricula for health care workers in Tanzania. As a result, a competency-based curricula was developed for pharmacists, pharmacy technicians, clinical officers and nursing cadres. An initiative to train pharmacy graduates in SCM as part of their professional training was thereafter embarked on in 2013.

## Method

Consultative meetings with various government and health education related institutions resulted in great enthusiasm and built momentum for creation of a pre-service training (PST) curriculum; an effective way of introducing principles and practices of health commodities SCM. Pilot orientation training was provided to 19 lecturers to teach health institutions how and where SCM can be integrated in PST. The acquired skillset enabled them to start teaching SCM in the coming semesters.

## Results

SCM was successfully integrated in the Bachelor of Pharmacy curriculum at the Muhimbili University College of Health Sciences (MUHAS) and the Institute of Health and Allied Sciences (IHAS). A total of 40 pharmacists and 35 pharmaceutical technicians completed training in SCM in 2013. An increasing interest in other public and private training institutions has been realized and currently SCMS is collaborating with St. Luke Foundation at the Kilimanjaro Christian Medical Centres School of Pharmacy and St. John University in Dodoma to integrate SCM in their Diploma of Pharmaceutical Technician’s course and Bachelor of Pharmacy course.

## Discussion

Education has been referred to as an effective “social vaccine” to curb the spread and ensure effective management of HIV/AIDS and other major diseases like Malaria and Tuberculosis. Having a knowledgeable human resource pool is paramount to obtaining accurate and timely logistics data to ensure health commodity security, effective and sustainable supply chains in Tanzania. The successful integration of PST is just one step in assuring future commodity security in Tanzania. A monitoring framework focusing on direct performance of pre- versus in -service training is necessary to fully realize the impact of the intervention.

## Lessons learned

Multidisciplinary involvement of government institutions is necessary in ensuring changes in curricula are accepted by all stakeholders and follow government policies and procedures. For this project to have a larger impact the right strategies, policies and plans must be in place for the recruitment and retention of SC workforce and professionalization of SCM.

